# Impact of Dietary Intake and Cardiorespiratory Fitness on Glycemic Variability in Adolescents: An Observational Study

**DOI:** 10.1016/j.cdnut.2025.104547

**Published:** 2025-01-21

**Authors:** Mingliang Ge, Stephanie R Lebby, Shivani Chowkwale, Caleb Harrison, Grace M Palmer, Keith J Loud, Diane Gilbert-Diamond, Mary Ellen Vajravelu, Jennifer L Meijer

**Affiliations:** 1 Department of Epidemiology, Geisel School of Medicine, Dartmouth College, Hanover, NH, United States; 2 Section of Obesity Medicine, Center for Digestive Health, Dartmouth Hitchcock Medical Center, Lebanon, NH, United States; 3 Center for Pediatric Research in Obesity and Metabolism and Division of Pediatric Endocrinology and Diabetes, University of Pittsburgh School of Medicine, Pittsburgh, PA, United States; 4 UPMC Children’s Hospital of Pittsburgh, Pittsburgh, PA, United States; 5 Department of Pediatrics, Geisel School of Medicine, Dartmouth College, Hanover, NH, United States; 6 Department of Medicine, Geisel School of Medicine, Dartmouth College, Hanover, NH, United States

**Keywords:** continuous glucose monitoring, 24-h dietary recalls, acute response, adolescents, maximum oxygen consumption, physical activity

## Abstract

**Background:**

Cardiorespiratory fitness (CRF), estimated by maximum oxygen consumption (VO_2_ max) during exercise, is worsening among adolescents and associated with a decline in metabolic health into adulthood. Glycemic patterns may provide a mechanism between CRF and health.

**Objectives:**

This study assessed the feasibility of measuring glycemic patterns using continuous glucose monitoring (CGM) in adolescents, aged 14–22 y, to estimate the relationship between VO_2_ max and glucose patterns.

**Methods:**

Healthy adolescents (*n* = 30) were recruited for a treadmill VO_2_ max test and to complete the following activities for 7–10 d: *1*) wear a Dexcom G6 CGM, *2*) complete ≥3 24-h dietary recalls, and *3*) complete 1 at-home oral glucose tolerance test (OGTT, 75 g glucose). Glycemic patterns were extracted as mean glucose, the coefficient of variance, the mean amplitude of glycemic excursions, and the mean of daily differences. The 2-h glucose responses to the OGTT and individual meals were extracted. Statistical analyses evaluated the relationship between VO_2_ max and *1*) overall glycemic patterns and *2*) the maximum glucose level and AUC response to OGTT and meals, stratified by sex.

**Results:**

Participant feasibility demonstrated that 90% completed CGM data (*n* = 27), 87% ≥7 d of CGM data (*n* = 26), 97% attempted OGTT (*n* = 29), and 93% completed ≥3 dietary recalls (*n* = 28). Most participants had normal BMI (70%) with an even distribution of sex (44% male). Males exhibited an inverse relationship between VO_2_ max and overall mean glucose (ß= –7.7, *P* = 0.04). Males demonstrated an inverse relationship between VO_2_ max and *1*) maximum glucose (ß = –29, *P* = 0.006) and AUC (ß = –2702, *P* = 0.001) in response to the OGTT and *2*) AUC (ß = –1293, *P* = 0.03) in response to meals. No association was observed between VO_2_ max and glucose patterns in females.

**Conclusions:**

A sex-specific relationship between VO_2_ max and glycemic patterns was observed, suggesting a unique metabolic capacity during late adolescence by sex.

This trial was registered at clinicaltrials.gov as NCT05845827.

## Introduction

Cardiorespiratory fitness (CRF), also known as “exercise capacity,” is the capacity of respiratory and circulatory systems to supply oxygen to skeletal muscle and to utilize the oxygen during exercise for the generation of energy. CRF is supported by lung capacity, capillary density, cardiac output, hemoglobin concentration, and mitochondrial function. CRF is estimated by measuring maximum oxygen consumption (VO_2_ max) during a graded exercise test that is typically performed on a treadmill or ergometer. There is an intrinsic aspect of exercise capacity, with an estimated 59%–72% of CRF being heritable [1], with strong influences by age and sex [2,3]. However, CRF can be altered through lifestyle modifications. For instance, improvements to VO_2_ max can be made with interval and high-intensity training, yielding 15%–20% increases in VO_2_ max [4]. Nevertheless, over the past several decades, the mean VO_2_ max has decreased in adolescents in the United States, with estimates showing that only 40% of adolescents have a healthy CRF [5]. This has clinical implications as lower VO_2_ max in childhood is associated with poorer health outcomes in adulthood, such as increased incidence of stroke [6], heart failure [7], type 2 diabetes (T2D) [8], cardiovascular disease (CVD) [9], and premature mortality [10]. Clarifying the mechanisms underlying the observed association between exercise capacity in childhood and metabolic health could elucidate targets for lifestyle and medical intervention.

Damaged mitochondrial function may provide a mechanistic understanding of the relationship between lower CRF and worsening metabolic health. We hypothesize that a high VO_2_ max is associated with more efficient macronutrient utilization, as enhanced mitochondrial metabolism is protective of metabolic health. This hypothesis was supported by studies that measured the metabolism of macronutrients utilizing a rat model of high compared with low CRF [11]. High-CRF rats have increased total energy expenditure, nonresting energy expenditure, and skeletal muscle mitochondrial coupling [4]. Low-CRF rats have increased hepatic lipid accumulation, decreased whole body lipid utilization, and decreased complete fatty acid metabolism [12]. Morris et al. [13] expanded on these results demonstrating that VO_2_ max is associated with postprandial fluctuations in the lipidome in humans in response to a lipid tolerance test. These studies suggest a positive association between VO_2_ max and fuel utilization, specifically fatty acid oxidation, via collection of blood samples in the clinical laboratory in response to acute challenges. Challenges arise when measuring mitochondrial function in humans in real-world settings because of limitations in technology to deliver real-time metabolism measurements [14,15]. Continuous glucose monitoring (CGM) provides an upstream metabolite of mitochondrial and lipid metabolism, allowing for individuals to be identified with metabolic dysregulation before additional blood biomarker assessment occurring in a clinical setting [15].

The overall objective of this feasibility study was to estimate the relationship between VO_2_ max and glycemic patterns using CGM in adolescents. Research in adults has shown that higher VO_2_ max is associated with better glycemic control, measured by hemoglobin A1c (HbA1c) [16] and 2-h postprandial glucose levels in response to a 75-g oral glucose tolerance test (OGTT) [17]. However, there have been no studies that analyzed the relationship between exercise capacity and glycemic control in adolescents, which provides an upstream estimate of mitochondrial dysfunction outside of the clinical laboratory. The results of this study may elucidate mechanisms between lower VO_2_ max in childhood and metabolic health in adulthood [[6], [7], [8], [9], [10]]. As part of a pilot trial, this study aimed to *1*) test for the feasibility of study design (Feasibility Aim), *2*) determine if VO_2_ max is associated with overall glycemic patterns (Aim 1), and *3*) determine if VO_2_ max is associated with the acute glucose response to an at-home OGTT and self-reported dietary intake (Aim 2). This study may lead to larger trials testing how VO_2_ max is related to the glycemic control of a variety of meal challenges with different macronutrient composition, potentially yielding prescriptive dietary recommendations based on exercise capacity.

## Methods

### Study cohort

Adolescents, aged 14–22 y, were recruited at Dartmouth Hitchcock Medical Center. Recruitment occurred via flyers distributed electronically and physically, specifically recruiting from the Dartmouth Hitchcock Medical Center pediatric clinic and list-servs. All individuals who expressed interest were screened for the following exclusion criteria: *1*) previous diagnosis of type 1 or 2 diabetes, *2*) previous diagnosis of hyperlipidemia, *3*) medications known to influence glucose metabolism (for example, metformin), *4*) inability to participate in a maximal exercise test on a treadmill, and *5*) inability to speak and/or write in English. For participants <18 y, consent was obtained from 1 parent and assent was obtained from the participant via a telephone appointment before the first study visit. For participants ≥18 y, consent was obtained from the participant via a telephone appointment before the first study visit. Study team members were available during the consent call to answer any questions, although using the teach-back method of consent to ensure that the parent and the participant understood the breadth of the study. Participants received gift card incentives for completion of the study, with a maximum of $225 provided for completing all the tasks. Only study team members had access to information collected during the study, with data stored behind institutional or equivalently approved firewalls. The study was monitored by the Dartmouth Health Human Research Protection Program (STUDY02001962) and registered on clinicaltrials.gov (NCT05845827). Study procedures took place between June and September 2023.

### Study design

**FIGURE 1:**
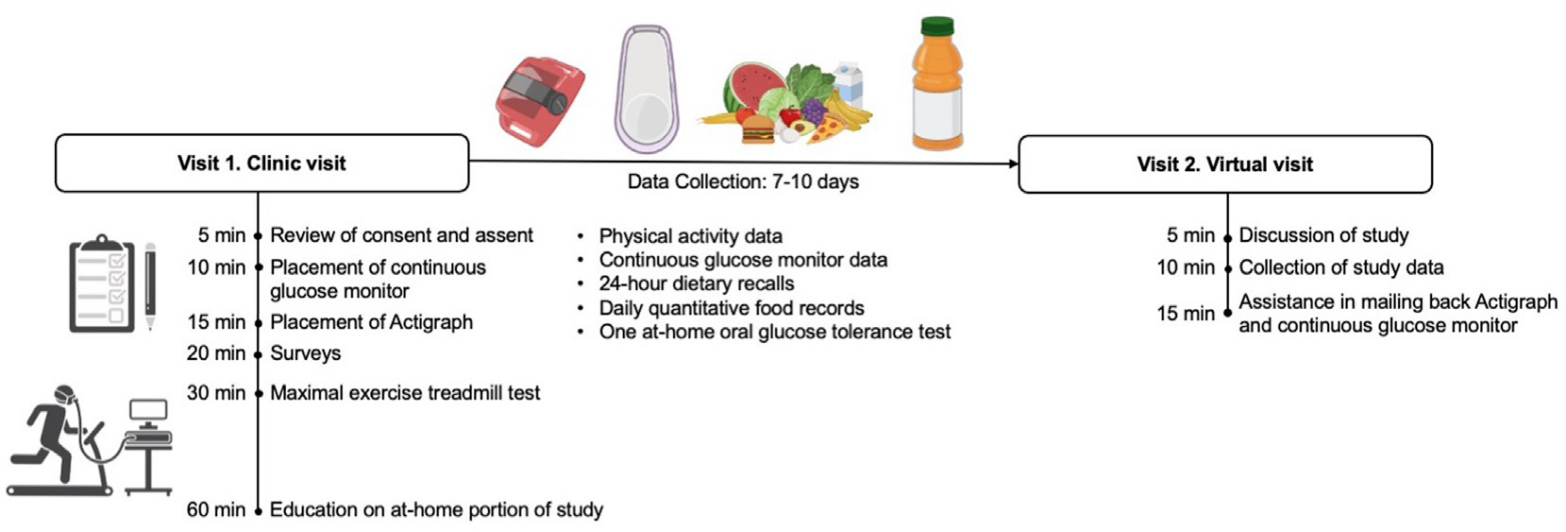
Study design. Participants arrived at the Nutrition and Metabolism Laboratory at Dartmouth Hitchcock Medical Center for Visit 1. Between Visits 1 and 2, all participants wore an Actigraph and Dexcom G6 continuous glucose monitor while recording daily dietary intake. Participants received 24-h dietary recalls via telephone. Participants completed an at-home oral glucose tolerance test. Participants completed the second visit virtually to close the study.

The study design is described in Figure 1. Participants arrived at the clinical visit at the Nutrition and Metabolism Lab (Visit 1) in comfortable clothing and sneakers for exercise after a 2 h fast to prevent sickness while running on the treadmill. All participants <18 y were accompanied by a parent or legal guardian. Visit 1 was ∼1 to 1.5 h. All participants returned for a virtual telehealth visit (Visit 2), which took place 7–10 d after the initial clinical visit. Visit 2 was ∼20 min.

### Anthropometrics and surveys

A trained study coordinator collected weight using Seca mBCA 554 scale and height using Seca 216 stadiometer, both collected on participants without heavy layers and without footwear. A trained study coordinator collected waist and hip circumference using the Seca 201 ergonomic circumference measuring tape following standard protocol. Participants answered surveys on demographics and family medical history, with participants <18 y receiving support from their parent or legal guardian. All participants self-reported pubertal stage using the Peterson Pubertal Development Scale [18], which stratifies development into prepubertal, early puberty, mid puberty, late puberty, and postpubertal.

### Maximal oxidative capacity treadmill protocol

Participants completed a maximal oxidative capacity treadmill test (VO_2_ max test), which was performed by a trained clinical exercise physiologist. The VO_2_ max test utilized the ParvoMedics TrueOne 2400 metabolic cart and the Trackmaster TMX428CP treadmill. The participant’s maximal oxidative capacity was estimated on the treadmill using protocols that vary the speed and grade of the treadmill with the objective of participant reaching volitional fatigue within 15 min. Our team replicated the British Columbia Children’s Hospital (BCCH) Protocol [19], which uses an incline at a constant 1% grade starting at a speed of 3.0 mph, increasing by 0.5 mph every minute until volitional fatigue. Additional details on clinical stress testing in the pediatric age group are provided by the American Heart Association [20].

The ParvoMedics TrueOne 2400 metabolic cart was used to measure oxygen consumption and carbon dioxide emission during the exercise protocol as participants breathed through a mouthpiece, which was connected by a breathing tube to the metabolic cart. Each mouthpiece was fitted to the participant’s mouth, and a nose plug was worn to prevent the participant from breathing through their nose. Participants wore a heart rate monitor around their chest to continuously monitor heart rate. Before starting the BCCH protocol, all participants had the opportunity to acclimate themselves to the treadmill, the mouthpiece, and the breathing tube. The treadmill can be controlled by the participant (for example, easily accessible “Stop” button and a rope that if pulled will stop the treadmill) and the study team. Participants had a 1-min warm-up at 3.0 mph with 0% incline. Participants exercised on the BCCH protocol until they claimed maximum exercise and exhaustion. All participants were asked to remain on the treadmill for a cool-down period of 2 min. All study team members provided the same level of encouragement to each participant to help them differentiate between exercise capacity constraints and lack of cooperation in exercise testing. The maximum respiratory exchange ratio (RER), the ratio between the metabolic production of carbon dioxide and the uptake of oxygen, ≥1.0 served as an indicator that the participant was nearing exhaustion, rather than stopping prematurely. The test was stopped immediately if the participant failed to conform to the protocol or if the participants experienced signs of discomfort, including increasing chest pain or fatigue, shortness of breath, wheezing, leg cramps, or claudication.

### Continuous glucose monitoring using Dexcom G6

Interstitial glucose levels were monitored between Visits 1 and 2 utilizing the Dexcom G6, which has been evaluated for accuracy in adolescents and adults with diabetes [21]. The Dexcom G6 was placed by the principal investigator of the study team on the back of the upper arm, with the left or the right arm selected by participant preference. The sensor measures interstitial glucose levels every 5 min for ≤10 d. Adhesive tape held the sensor in place; however, participants were educated on limiting the use of creams (for example, sunscreen) around the sensor to help its longevity. The participant was blinded to the glucose readings to prevent alterations in habits to modify glucose readings. Participants were asked to wear the CGM system for ≥7 d. Participants received a prelabeled envelop to mail the Dexcom G6 transmitter back to the study team after Visit 2. The study team transferred data from the Dexcom G6 transmitter to the Dexcom Clarity computer application via Bluetooth. Raw data were downloaded in comma seperated values (CSV )format and imported into R Studio.

### Habitual dietary intake utilizing 24-h dietary recalls

Dietary intake was evaluated by conducting 24-h dietary recalls utilizing the Nutritional Data System for Research (NDSR) software version 2023 developed by the Nutrition Coordinating Center (NCC), University of Minnesota, Minneapolis, MN, which leverages the multiple-pass method [22]. Three 24-h dietary recalls were collected with 2 on a weekday and 1 on the weekend between Visit 1 and Visit 2. To facilitate, at Visit 1, participants informed the study team times during the day when they would be available for the recall to increase the success rate of data collection. Participants were unaware of the date that they would receive a 24-h dietary recall to prevent alterations in dietary habits. As 24-h dietary recalls can be challenging in the adolescent population, our study design requested participants to complete dietary diaries each day between Visits 1 and 2, recording all foods and beverages consumed. The dietary diaries had a section to report the time of the meal and the quantity of each food or beverage consumed. Participants were asked if they had their dietary diaries nearby when receiving a phone call for a recall. The dietary diaries were returned to the study team in the prelabeled envelop after Visit 2. They were occasionally referenced in quality control of the 24-h dietary recalls in NDSR; however, the meals from the 24-h dietary recalls were the only meals extracted to assess the postprandial glucose response. All dietary data were reviewed by a registered dietitian.

Data were extracted from the NDSR at the meal level and the participant level. At the meal level, the amount of energy (kcal), carbohydrates (g), fat (g), protein (g), dietary fiber (g), sugars (g), and the fat-to-carbohydrate ratio were extracted. At the participant level, daily total energy (kcal) and the 2020 Healthy Eating Index (HEI) [23] were extracted. The 2020 HEI was created using the Statistical Analysis System (SAS) algorithm by the NCC, University of Minnesota, Minneapolis, MN and the National Cancer Institute. Each participant had their daily total energy and HEI averaged across all 24-h dietary recalls to create a participant mean.

### Standardized glucose tolerance test

Participants were asked to complete a standard OGTT at home after a 12-h overnight fast. The drink contained 75 g of glucose as a liquid solution from Fisher Scientific Company. Participants self-reported the date and time of taking the OGTT. They were instructed to not have anything to eat or drink for 2 h after drinking the OGTT. They were asked to refrain from moderate-to-vigorous exercise for 2-h after drinking the OGTT.

### Actigraph

Participants were asked to wear an ActiGraph GT9X triaxial accelerometer (Pensacola, FL) between Visits 1 and 2 to objectively assess habitual physical activity (PA). This accelerometer was chosen as it has shown to be a reliable and valid measure of habitual PA in children with established data acquisition and processing methods [24]. Participants wore the monitor on their nondominant wrist during all waking hours. Participants were asked to remove the monitor during periods of sleeping, bathing, or swimming, as well as contact sports. Participants completed accelerometer logs, noting the time when the device was put on and taken off. Actigraphs were returned to the study team in the prelabeled envelop after Visit 2. Upon collection, data were exported from the device using ActiLife software, Version 16.3.3 (ActiGraph). The GT9X accelerometer generates a variable output voltage signal, proportional to acceleration in 3 orthogonal places (vertical, anteroposterior, and mediolateral). Acceleration is converted to activity counts and stored on the device.

### Statistical analysis

Our feasibility goal was subdivided to guide refinement for future implementation, including the proportion of participants who: *1*) completed the treadmill VO_2_ max test with an RER ≥ 1.0, *2*) returned the CGM transmitter to the study team with valid data, *3*) wore the CGM system for ≥ 7 d, *4*) attempted the at-home OGTT, *5*) completed the at-home OGTT without issue (for example, issue with time report) or adverse reaction (for example, vomiting), and *6*) reported 2 weekday and 1 weekend 24-h recalls. Of these feasibility goals, only returning the CGM transmitter to the study team with valid data was utilized for exclusion criteria.

Participant demographics and lifestyle characteristics were reported as mean and SD for continuous variables and count (percentage) for categorical variables. Sex differences in variables were assessed using Fisher’s exact tests for categorical variables [25] and Wilcoxon rank-sum tests for continuous variables [26]. Spearman correlations were utilized to assess for associations between anthropometrics, exercise capacity, glycemic patterns, dietary intake, and PA, with the objective of determining which covariates to include into the statistical models. Select variables were reported as histograms to demonstrate distribution.

Data processing included the following: For CGM, false glucose readings (0.43% of total readings) were removed, including non-numerical values (“LOW”), extremely low values (<50 mg/dL), and missing values (“NA”). R package iglu (version 4.1.6) [27,28] was used to calculate continuous-glucose-monitor-derived metrics, including the mean glucose level across all readings (mean), coefficient of variation (CV), mean amplitude of glycemic excursions (MAGE) [29], and mean of daily difference (MODD) [30]. Metrics were computed using the package’s all_metrics() function. For Actigraph analyses, raw data (GT3X files) were downloaded from ActiLife and processed using the GGIR package (Version 3.0–0) in R [31]. GGIR processing first included autocalibration of raw signals, identification of nonwear, and conversion to gravity-corrected vector magnitude units (Euclidean Norm Minus One—ENMO) over 5-s epochs [32,33]. By default, GGIR imputed nonwear data using the mean at similar time points on other days of the study period. To determine PA levels, previously published youth-specific ENMO cut-points of sedentary behavior (< 35.6 mg), light physical activity (≥35.6–201.4 mg), moderate physical activity (MPA) (≥201.4–707.0 mg), and vigorous physical activity (VPA) (≥707.0 mg) were used [34,35]. The default sleep algorithm (HDCZA) was used to identify the sleep period time, where sustained inactivity was assumed to represent sleep [36,37]. The weighted mean (mean across all days where weekend days are weighted 2/5 relative to the contribution of weekdays) number of minutes spent in each level of PA, as well as sleep, was extracted for use. Moderate-to-vigorous physical activity (MVPA) was calculated as adding MPA + VPA. One participant did not have Actigraph data collected due to a shortage of Actigraphs in the laboratory. For dietary data, data were described per meal and/or as an mean per participant. Macronutrient distribution per meal was described using a ternary diagram using the *ggtern* package (Version 2.2.2) within ggplot2 in R.

Aim 1 was to determine if exercise capacity, measured using VO_2_ max, is associated with overall glycemic patterns. The predictor, VO_2_ max (L/min), was not adjusted for weight in analyses to prevent collinearity in the independent variables, as the Pearson’s correlation between VO_2_ max and weight (kg) was 0.71. Our outcomes were glucose mean, CV, MAGE, and MODD. For multiple linear regression analyses, the R *lm()* function was used to determine the influence of VO_2_ max (L/min) on iGlu metrics, utilizing the following models: VO_2_ max (L/min) and HEI (Model 1, *n* = 27); VO_2_ max (L/min) and MVPA (Model 2, *n* = 26); VO_2_ max (L/min) and age (Model 3, *n* = 27); and VO_2_ max (L/min) and BMI (Model 4, *n* = 27). All models were stratified by sex due to established sex differences in exercise capacity starting at puberty [38].

Aim 2 was to determine if exercise capacity, measured using VO_2_ max, is associated with the acute glucose response to an OGTT and the acute glucose response to self-reported dietary intake.

Glucose response to the OGTT was measured using CGM, which recorded glucose readings every 5 min for 2 h post-OGTT. Timepoint 0 was the first glucose measurement after the participant self-reported consuming the OGTT. Timepoint 120 was the first glucose measurement 120 min after timepoint 0. The maximum glucose level (mg/dL) was the highest glucose level reported between timepoints 0 and 120. The AUC of glucose response was calculated utilizing the trapezoidal rule [39], accounting for the entire area, down to glucose of 0 mg/dL. For multiple linear regression analyses, the R *lm()* function was used to determine the influence of VO_2_ max (L/min) on *1*) max glucose and *2*) AUC glucose, utilizing the following models: VO_2_ max (L/min) and HEI (Model 1, *n* = 25); VO_2_ max (L/min) and MVPA (Model 2, *n* = 24); VO_2_ max (L/min) and age (Model 3, *n* = 25); and VO_2_ max (L/min) and BMI (Model 4, *n* = 25). All models were stratified by sex.

Glucose response to self-reported meals was determined using the 24-h dietary recalls. Maximum glucose level and AUC glucose were calculated as done in response to the OGTT. There were 625 meals collected (*n* = 27), with 412 meals that were collected while the participant was wearing the continuous glucose monitor for ≥2 h. Meals were excluded from analysis if they overlapped with another meal within a 2-h period (181 meals) or had excessive “NA” glucose readings (1 meal). Excluding overlapping meals was crucial to prevent potential confounding effects where glucose responses from multiple meals might be erroneously attributed to a single meal. After exclusion, 230 meals were analyzed. Linear mixed-effect models were used with the individual as a random effect due to each participant having >1 meal for analysis. The R *lme4* package (version 1.1–35.5) was used to determine the influence of VO_2_ max (L/min) on *1*) max glucose and *2*) AUC glucose, utilizing the following models: VO_2_ max (L/min) and HEI (Model 1, *n* = 27); VO_2_ max (L/min) and MVPA (Model 2, *n* = 26); VO_2_ max (L/min) and age (Model 3, *n* = 27); and VO_2_ max (L/min) and BMI (Model 4, *n* = 27). A fourth model was considered to account for individual meal composition. Meal composition was captured using principal component analysis (PCA) with the following meal variables: amount of energy (kcal), carbohydrates (g), fat (g), protein (g), dietary fiber (g), sugars (g), and the fat-to-carbohydrate ratio. PCA was conducted using the prcomp() function (version 4.4.1) with data centering and scaling applied. Both PC1 and PC2 were retained (Supplemental Figure 1). Model 5 was VO_2_ max (L/min), meal composition PC1, and meal composition PC2 (Model 5, *n* = 27). All models were stratified by sex.

For all analyses, an unadjusted *P* value < 0.05 was considered significant. All data analysis was conducted using the R Language for Statistical Computing (R Core Team. R: A Language and Environment for Statistical Computing. Available Online: http://www.R-project.org/ (accessed from September 2023 through August 2024)).

## Results

A total of 30 participants completed Study Visit 1 in clinic and Study Visit 2 via telehealth (Figure 1). 100% of participants completed the treadmill VO_2_ max test with an RER ≥ 1.0 (*n* = 30). 93% of participants returned the CGM transmitter to the study team with valid data (*n* = 27), with missingness due to inability to sense glucose levels from a placement issue (*n* = 2) and not returning the CGM transmitter (*n* = 1). 87% of participants had ≥7 d of CGM data (*n* = 26), due to invalid CGM data (*n* = 3, see previous sentence) and sweat causing the CGM sensor to fall off (*n* = 1). 97% of participants attempted the at-home OGTT (*n* = 29), with 1 participant not attempting due to the CGM sensor falling off on day 6. 83% of participants had a valid at-home OGTT with complete CGM data (*n* = 25), because of invalid CGM data (*n* = 3), not reporting the time of the OGTT (*n* = 1), and not being fasted (*n* = 1). 93% of participants completed three 24-h recalls with 2 recalls on a weekday and 1 recall on a weekend (*n* = 28). Of these feasibility goals, only returning the CGM transmitter to the study team with valid data was utilized for exclusion criteria (*n* = 27, Supplemental Figure 2).

The study cohort demographics and lifestyle characteristics are reported in Table 1 (*n* = 27). There was an approximately even distribution of age (44% <18 y) and sex (44% male), with most participants identifying as white (85%). Most participants self-reported late and postpubertal stages (92%). Nineteen participants were of normal BMI (70%), 5 participants were overweight (19%), and 2 participants were obese (7%). The overall mean BMI was 23.4 kg/m^2^ and the overall mean waist-to-hip ratio was 0.80. BMI and waist-to-hip ratio had a mild correlation (Spearman correlation r = 0.52), with a stronger correlation in females (r = 0.76) than males (r = 0.54) (Supplemental Figure 3). Most participants had a high maximal exercise capacity classified as the highest tertial of VO_2_ max percentile (81%), with an mean VO_2_ max of 3.34 L/min. Sixteen participants had over 30 min of MVPA daily from Actigraph counts (59%). Fourteen participants reported over 2000 kcals consumed per day (52%), with a cohort mean HEI score of 50. Distributions of lifestyle characteristics are visualized in Supplemental Figure 4 with mean values reported in Supplemental Table 1, by sex. Females within the study cohort were significantly older, within later pubertal stages, and consumed a healthier diet as measured by the HEI score (Supplemental Table 1). Males within the study cohort had a higher VO_2_ max, with no sex differences in VO_2_ max percentile, and consumed more calories (Supplemental Table 1).

**TABLE 1:** Demographics and lifestyle characteristics of study participants (*n* = 27).

Characteristic	n	%
Age (y)		
<18	12	44%
≥18	15	56%
Sex		
Male	12	56%
Female	15	44%
Crockett pubertal development score		
Prepubertal	1	4%
Early puberty	0	0%
Mid puberty	1	4%
Late puberty	12	44%
Postpubertal	13	48%
BMI (kg/m^2^)		
<18.5	1	4%
18.5–24.9	19	70%
25.0–29.9	5	19%
≥30.0	2	7%
Maximal exercise capacity percentile		
VO_2_ max percentile <33%	3	11%
VO_2_ max percentile 33%–66%	2	7%
VO_2_ max percentile ≥66%	22	81%
Energy intake (mean)		
<1500 kcal per d	3	11%
1500–2000 kcal per d	8	30%
≥2000 kcal per d	14	52%
Moderate-to-vigorous physical activity (mean, weighted)		
<30 min per day	10	37%
30–60 min per day	10	37%
≥60 min per day	6	22%

**FIGURE 2:**
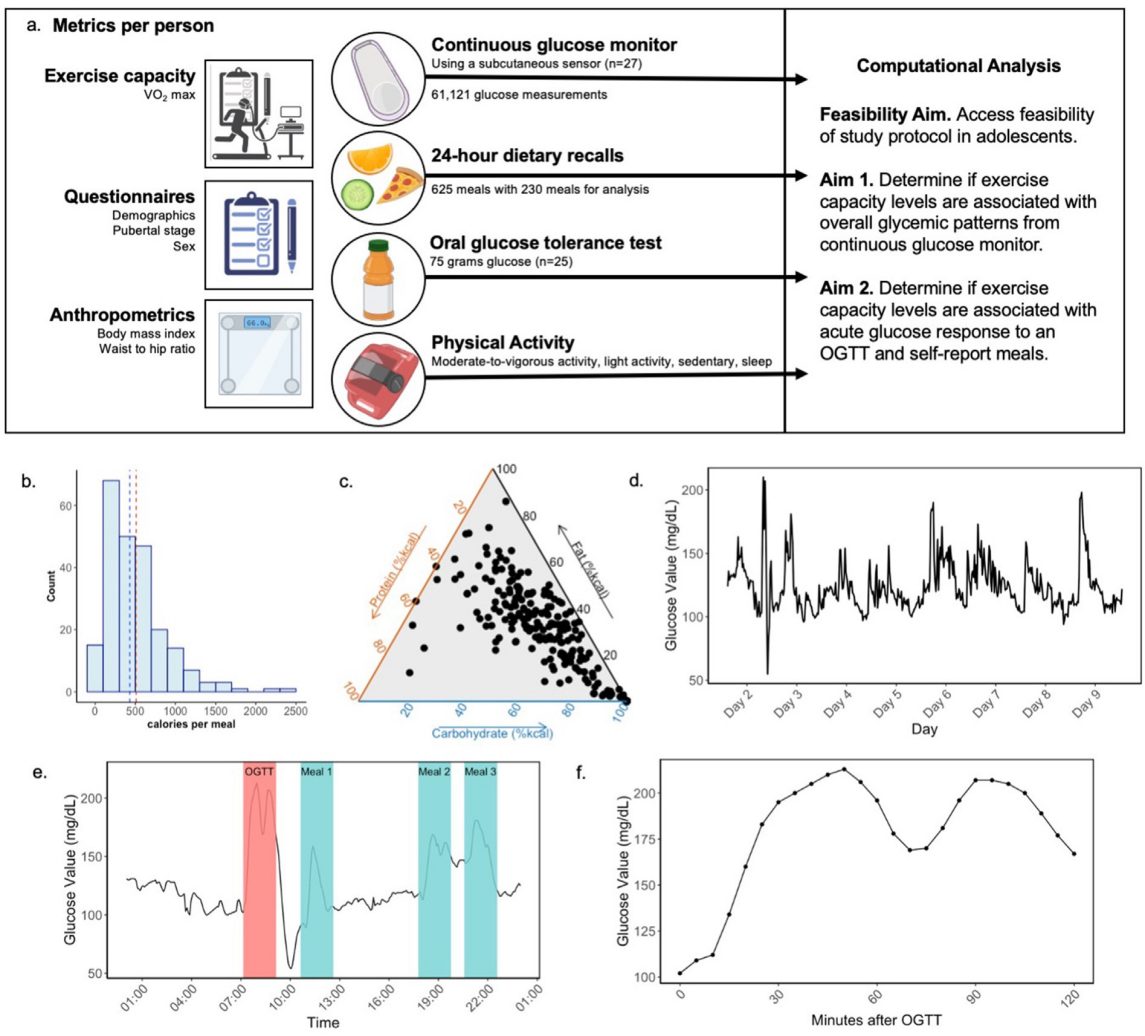
Profiling the postprandial glycemic response to at home glucose tolerance test and habitual dietary intake. (a) Study parameters collected per person to complete the computational analysis goals. Participants reported 24-h dietary recalls, presenting 230 meals with (b) calories per meal with mean (red line) and median (blue line) described and (c) the percentage of calories from protein, fat, and carbohydrates. (d) Glycemic variability across all days wearing the continuous glucose monitor is demonstrated for Participant 016, (e) demonstrating the glucose response on Day 2, highlighting the time of the at-home oral glucose tolerance test (OGTT) and self-reported meals. (f) Participant 016 demonstrated a 2-h biphasic response to the at-home OGTT.

Metrics collected per person during the study protocol are described in Figure 2A. The dataset included a total of 61,271 glucose readings across all participants. The glucose response was extracted post 230 meals (see methods). Energy content of the meals is reported in Figure 2B, with the mean of 509 calories per meal, and percent of calories from carbohydrates, fat, and protein is reported in Figure 2C. The integration of glucose measurements, the at-home glucose tolerance test, and self-reported dietary intake is demonstrated for Participant 016 in Figure 2D–F. Participant 016 wore the CGM for 9 d with overall glucose metrics reported in Figure 2D. On Day 2, the participant completed the at-home glucose tolerance test at 7:08 am (self-reported) (Figure 2E); the extracted 2-h postprandial response to the 75 g glucose solution is shown (Figure 2F). On Day 2, participant 016 self-reported meals at 10:37 am, 5:46 pm, and 8:34 pm via 24-h dietary recalls; the extracted 2-h postprandial response is shown (Figure 2E).

### Association between exercise capacity and overall glycemic patterns

**FIGURE 3:**
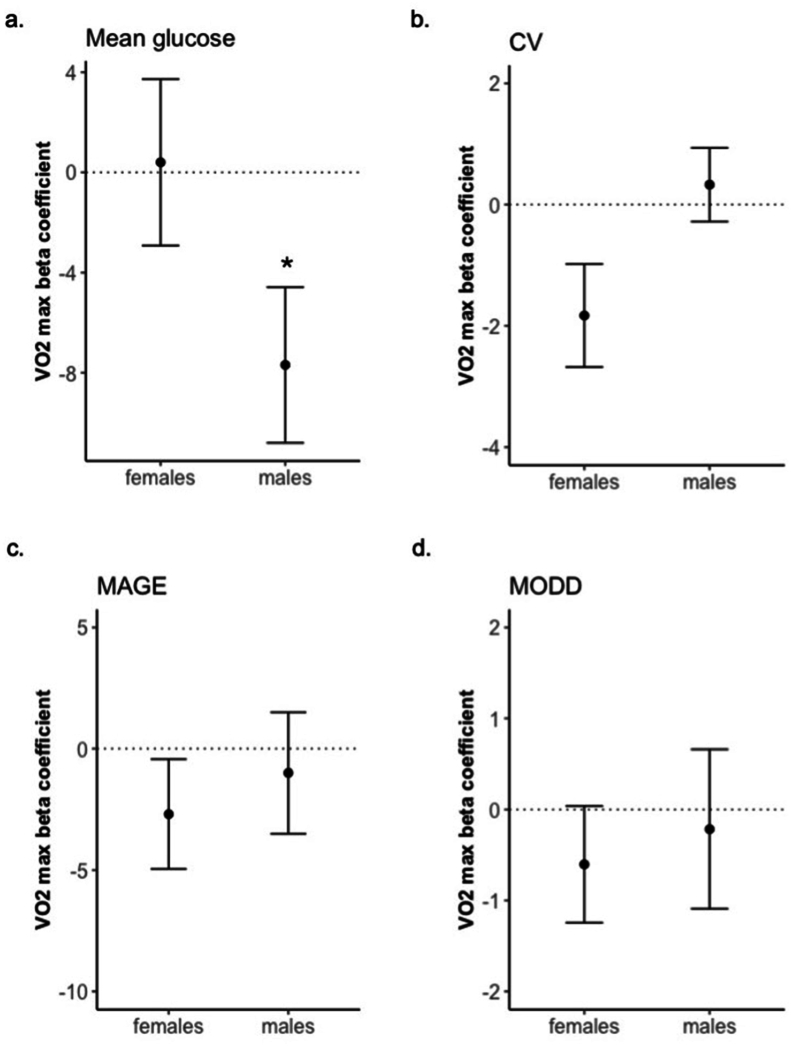
Relationship between exercise capacity with overall glycemic variability, stratified by sex. Plots demonstrate beta coefficients from model of VO_2_ max + overall healthy eating index association with iGlu metrics, in males (*n* = 12) and females (*n* = 15). (a) Mean glucose levels across all continuous glucose monitor measures; (b) coefficient of variance across all continuous glucose monitor measures; (c) mean amplitude of glycemic excursions; and (d) mean of daily differences. All models stratified by sex with error bars describing standard error of beta coefficient. “∗” *P* value < 0.05.

Aim 1 was to demonstrate the relationship between VO_2_ max (L/min) with overall glycemic patterns, measured using mean, CV, MAGE, and MODD (Supplemental Figure 5). All models were sex-stratified because of the sex differences in performance [38] as demonstrated by differences in VO_2_ max in our cohort (male = 3.91 L/min compared with female = 2.89 L/min, *P* = 0.0004, Supplemental Table 1). Males demonstrated a higher mean glucose level than females (male = 121 mg/dL compared with female = 106 mg/dL, *P* = 0.001, Supplemental Table 1). Females demonstrated a higher glucose CV than males (male = 13.6% compared with female = 16.7%, *P* = 0.001, Supplemental Table 1). Beta coefficients describing the relationship between VO_2_ max (L/min) with glycemic patterns, adjusting for HEI (Model 1), are reported in Figure 3. In males, VO_2_ max was inversely associated with mean glucose level (ß = –7.7, *P* = 0.035, Figure 3A), with beta coefficients similar when adjusting for MVPA (Model 2), age (Model 3), or BMI (Model 4) (Supplemental Table 2). In females, VO_2_ max was trending to an inverse association with CV (ß = –1.8, *P* = 0.052, Figure 3B), decreasing in strength when including MVPA (Model 2), age (Model 3), or BMI (Model 4) in the models. Although in females glycemic patterns were not associated with VO_2_ max, BMI was inversely associated with MAGE (ß = –1.365, *P* = 0.011, Supplemental Table 2) and MODD (ß = –0.411, *P* = 0.025, Supplemental Table 2).

### Association between exercise capacity and 2-h glucose response to an oral glucose tolerance test

**FIGURE 4:**
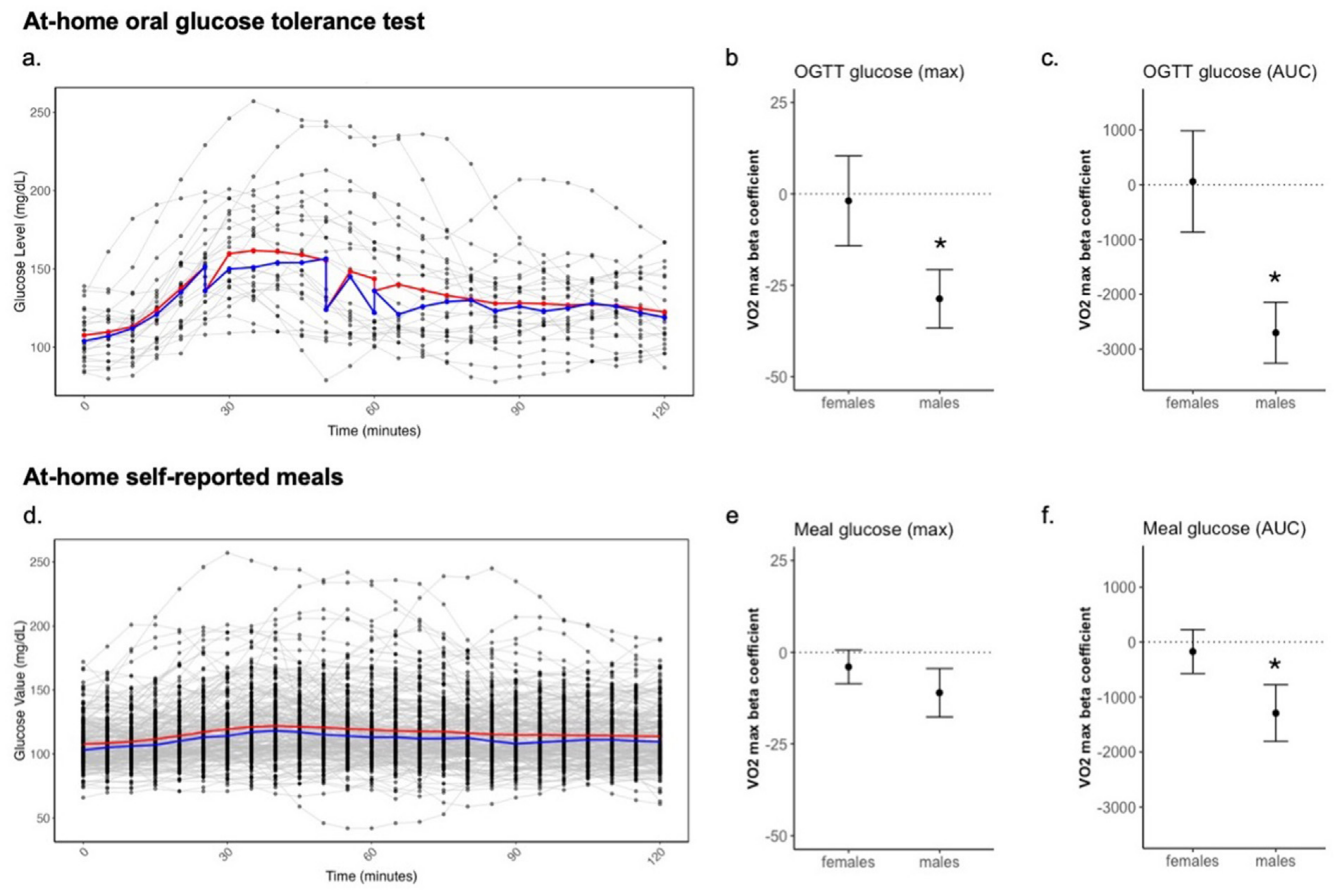
Relationship between exercise capacity with acute glucose response to an at-home oral glucose tolerance test and self-reported meals, stratified by sex. (a) The individual glucose response to an at-home OGTT (75 g of glucose) across 120-min with mean (red line) and median (blue line) described. Plots demonstrate beta coefficients from model of VO_2_ max + overall healthy eating index association with (b) maximum glucose in response to the OGTT and (c) AUC in response to the OGTT, in males (*n* = 12) and females (*n* = 13). (d) The individual glucose response to meals reported (230 meals) across 120 min with mean (red line) and median (blue line) described. Plots demonstrate beta coefficients from model of VO_2_ max + overall healthy eating index association with (e) maximum glucose in response to the meal and (f) AUC in response to the meal, in males (*n* = 12) and females (*n* = 15). All models stratified by sex with error bars describing standard error of beta coefficient. “∗” *P* value < 0.05.

Aim 2, part 1 was to demonstrate the relationship between VO_2_ max (L/min) with the acute glucose response to an at-home OGTT, measured using maximum glucose level and glucose AUC (Supplemental Figure 6). Individual 2-h glucose response to the OGTT is shown in Figure 4A. No sex differences were observed in the maximum glucose level and glucose AUC (Supplemental Table 1). Beta coefficients describing the relationship between VO_2_ max (L/min) and maximum glucose or glucose AUC, adjusting for HEI, are reported in Figure 4B and 4C, respectively. In males, VO_2_ max was inversely associated with maximum glucose level (ß = –28.7, *P* = 0.006, Figure 4B) and with glucose AUC (ß = –2702, *P* = 0.001, Figure 4C). Both associations in males increased in strength when adding age into the model (Supplemental Table 3). No associations were observed in females (Supplemental Table 3).

### Association between exercise capacity and 2-h glucose response to self-reported meals

Aim 2, part 2 was to demonstrate the relationship between VO_2_ max (L/min) with the acute glucose response to self-reported meals, measured using maximum glucose level and glucose AUC (Supplemental Figure 7). Individual 2-h glucose response to 230 self-reported meals is shown in Figure 4D. Males demonstrated a higher maximum postprandial glucose (male = 149 mg/dL compared with female = 132 mg/dL, *P* < 0.001, Supplemental Table 1) and a larger glucose AUC (male = 14879 compared with female = 13226, *P* < 0.001, Supplemental Table 1) than females. Beta coefficients describing the relationship between VO_2_ max (L/min) and maximum glucose or glucose AUC, adjusting for HEI, are reported in Figure 4E and 4F, respectively. There was no significant association between VO_2_ max and maximum glucose level in response to meals (Model 1, Figure 4E). When adjusting for BMI, the association between VO_2_ max and maximum glucose level in response to meals reaches significance (ß = –15.899, *P* = 0.038, Supplemental Table 4). In males, VO_2_ max was inversely associated with glucose AUC (ß = –1293, *P* = 0.033, Figure 4F). This association remained stable when adding MVPA, age, BMI, or meal composition PCs into the model (Supplemental Table 4). No associations were observed in females (Supplemental Table 4).

## Discussion

To our knowledge, the results of this study are the first to consider the relationship between exercise capacity and CGM-derived glucose metrics in healthy adolescent males and females, which may provide an early predictor of metabolic health. Prior studies have analyzed this relationship in adolescents with type 1 diabetes [40]; however, the predictive ability of these metrics in adolescents is unknown. The relationship between exercise capacity and glycemic control has been demonstrated in healthy adults. In a cohort of 313 heterogeneous adults, Solomon et al. demonstrated that a higher VO_2_ max is associated with lower HbA1c, lower fasting glucose, lower 2-h post-OGTT glucose, and higher insulin sensitivity [17]. These results were similarly replicated using VO_2_ peak and HbA1c in adults with overweight and obesity, without T2D [41]. For over a decade, it has been apparent that lower exercise capacity is an early marker of impaired insulin sensitivity in the development of insulin resistance and T2D [42]. However, to our knowledge, no studies have compared VO_2_ max with CGM-derived glucose metrics in healthy adolescents. The rationale of this study is to determine mechanisms underlying the development of metabolic diseases, such as T2D, at an earlier age. The long-term goal is to create prediction algorithms to classify the risk of developing disease during adolescence and early adulthood. The clinical application is to intervene to increase exercise capacity, as only 40% of adolescents have an “adequate” CRF for their age and sex [5].

Our study suggests that exercise capacity in males is inversely associated with acute and overall glucose utilization, independent of dietary quality, which is not observed in females. There were no differences in VO_2_ max percentile between males and females, suggesting that relative to sex, exercise capacity levels were similar. Our results may suggest differential mechanisms in the relationship between exercise capacity and glucose utilization by sex. One hypothesis is that excess muscle mass in males may be associated with heightened glucose control. Studies have demonstrated that males have overall greater skeletal muscle mass compared with females in general [43] and that skeletal muscle mass is responsible for 80% of oral glucose uptake and crucial for glucose clearance [44]. A limitation in our study is that we did not measure muscle mass to further this hypothesis. A second hypothesis is that differences in pubertal development and age may have confounded the association as females were significantly older and within later pubertal stages (Supplemental Table 1). Postpubertal estrogen levels are influential in energy balance and insulin response [45]. For example, compared with age-matched men, premenopausal women have enhanced insulin sensitivity normalized to lean mass [46]. Previous studies have reported women having lower fasting plasma glucose and higher 2-h plasma glucose in comparison with men [47]. Our null results in females may be suggestive of estrogen levels being a larger determinant in glucose control than exercise control. Future studies with larger sample sizes are warranted to elucidate these sex differences.

A novel factor in our study design was extracting the 2-h glucose response using CGMs to meals. The use of glycemic responses for personalized nutrition was established in 2015 by Zeevi et al [48], which extracted the postprandial glucose response to 46,898 real-life meals in an 800-person cohort. Several other studies used similar approached in adults [49,50]; however, to our knowledge, only 1 other study integrated self-reported meals with glycemic response in adolescents [51]. Future research and expert consensus must be reached on how to define a “meal” when studying the acute glycemic response. The length of glucose extracted from CGM measurements varied in previous studies, as Gonzalez-Rodriguez et al. extracted a 6-h response [49] and Fechner et al. extracted a 2-h response [50]. Neither of these studies considered if participants consumed meals immediately prior or after their extracted meal. A novelty of our study was removing any meals that overlapped during a 2-h period. A limitation in this approach was that 181 meals (44%) were removed from analysis. Nevertheless, our study demonstrates the feasibility of integrating the postprandial glucose response to meals in adolescents, yielding a better understanding of glycemic in free-living conditions as participants maintain their usual routine of meals and exercise. However, limitations are still apparent with classifying glucose response to meals using CGMs as a marker of metabolic health [52]. Future studies may consider other mitochondrial metabolites to measure in free-living environments [53].

Overall, given the small sample size with most participants (81%) having a high VO_2_ max level, these findings are preliminary and may not generalize to different populations and settings. Future studies should consider recruiting adolescents with a range of exercise capacity. A strength of this study was that it utilized gold-standard techniques for estimating CRF with a graded treadmill test and a trained exercise physiologist. This study examined glycemic in free-living conditions as participants maintain their usual routine of meals and exercise. A strength of this study was that it had a registered dietitian on the study team and combined 2 tools for measuring dietary intake, with the 24-h dietary recall as the self-reported gold standard and daily food diaries to help participant recall all that they consumed. Strong statistical analyses were used, considering mixed models to account for individual variation in meal responses. Our study demonstrated to be feasible with the adolescent population with several avenues for improvement in future research endeavors. Incentives should be increased for returning the CGM transmitter, with future studies considering an in-person Visit 2 rather than a remote visit. Emphasis should be placed on completing the at-home OGTT and 24-h recalls within days 2–6 of wearing the CGM due to issues with the CGM falling off, especially in the warmer months during summer. Considerations should be made with heightening the emphases of reporting the exact time the OGTT and meals were consumed for the accurate extraction of the 2-h postprandial response. For instance, Participant 004 reported consuming the OGTT at 9:00 am; however, their glucose levels did not rise until 9:26 am (Supplemental Figure 8). This example may be a report error or a physiological response; however, we cannot be certain based on only a written self-reported time. Future studies could consider adding a picture with a time stamp of when acute challenges and meals were consumed, as 35% of OGTTs and 45% of meals were reported at the exact hour or exact half-hour, potentially suggesting that participants were rounding their time reporting. Finally, larger sample sizes are crucial to conform and further elucidate these sex-specific relationships. Our study is limited to healthy adolescents toward the later stages of puberty.

In conclusion, this study provides valuable insights into the complex, sex-specific relationship between CRF and glucose metabolism in adolescents. These findings have important implications for tailoring metabolic health interventions and highlight the need for more nuanced, sex-specific approaches in both research and clinical practice. In clinical practice, these results present opportunities to integrate CGM data with dietary analyses, which is not available in many current practices. Future studies addressing the methodological considerations outlined above will be crucial in advancing our understanding of these intricate physiological relationships.

## Author contributions

The authors’ responsibilities were as follows – DGD, MEV, JLM: designed research: SRL, SC, GMP: conducted research; JLM: provided essential reagents or materials; MG, CH, JLM: analyzed data or performed statistical analysis; MG, SRL, GMP, JLM: wrote paper; MG, JLM: had primary responsibility for final content; and all authors: read and approved the final manuscript.

## Data availability

Data described in the manuscript, code book, and analytic code will be made available upon request pending (for example, application and approval, payment, other).

## Funding

This research was funded by the Department of Medicine Scholarship Enhancement in Academic Medicine Awards Program at Dartmouth Health and the Office of Research and Operations at Dartmouth Health.

## Conflict of interest

The authors report no conflicts of interest.
