# Supplementary material for: Impact of Dietary Intake and Cardiorespiratory Fitness on Glycemic Variability in Adolescents: An Observational Study
Source: Curr Dev Nutr. 2025 Jan 21;9(2):104547. doi: 10.1016/j.cdnut.2025.104547 (PMC11847740; doi:10.1016/j.cdnut.2025.104547)
Supplement: Multimedia component 2 [file mmc2.pdf]

TITLE.

Impact of dietary intake and cardiorespiratory fitness on glycemic variability in adolescents: an observational study.

AUTHORS.

Mingliang Ge, Stephanie R. Lebby, Shivani Chowkwale, Caleb Harrison, Grace M. Palmer, Keith J. Loud, Diane Gilbert-Diamond, Mary Ellen Vajravelu, Jennifer L. Meijer

SUPPLEMENTARY MATERIALS TITLES

Supplementary Figure 1. Defining meal composition variable using principal component analysis.

Supplementary Figure 2. Data availability within study cohort.

Supplementary Figure 3. Relationship between body mass index (BMI) and waist-to-hip ratio, stratified by sex.

Supplementary Figure 4. Histograms of physical health and lifestyle characteristics.

Supplementary Figure 5. Histograms of glucose metrics extracted from continuous glucose monitor.

Supplementary Figure 6. Histograms of glucose response to oral glucose tolerance test.

Supplementary Figure 7. Histograms of glucose response to individual meals.

Supplementary Figure 8. Individual response to the at home glucose tolerance test.

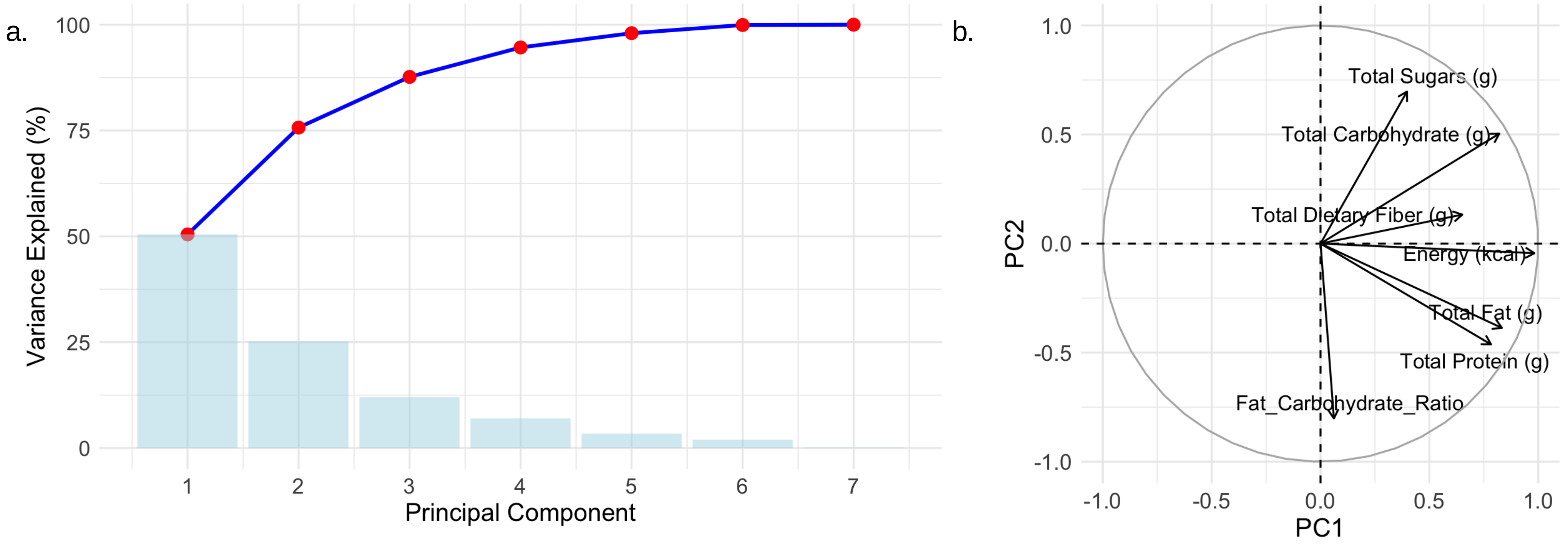

Supplementary Figure 1. Defining meal composition variable using principal component analysis. Meal variables imported into PCA included energy (kcal), total carbohydrates (g), total protein (g), total fat (g), total dietary fiber (g), total sugars (g), and fat to carbohydrates ratio. (a) Scree plot representing component variance (bars) and total variance explained (line, red dots**).** (b) Biplot demonstrating importance of meal variables within PC1 vs. PC2.

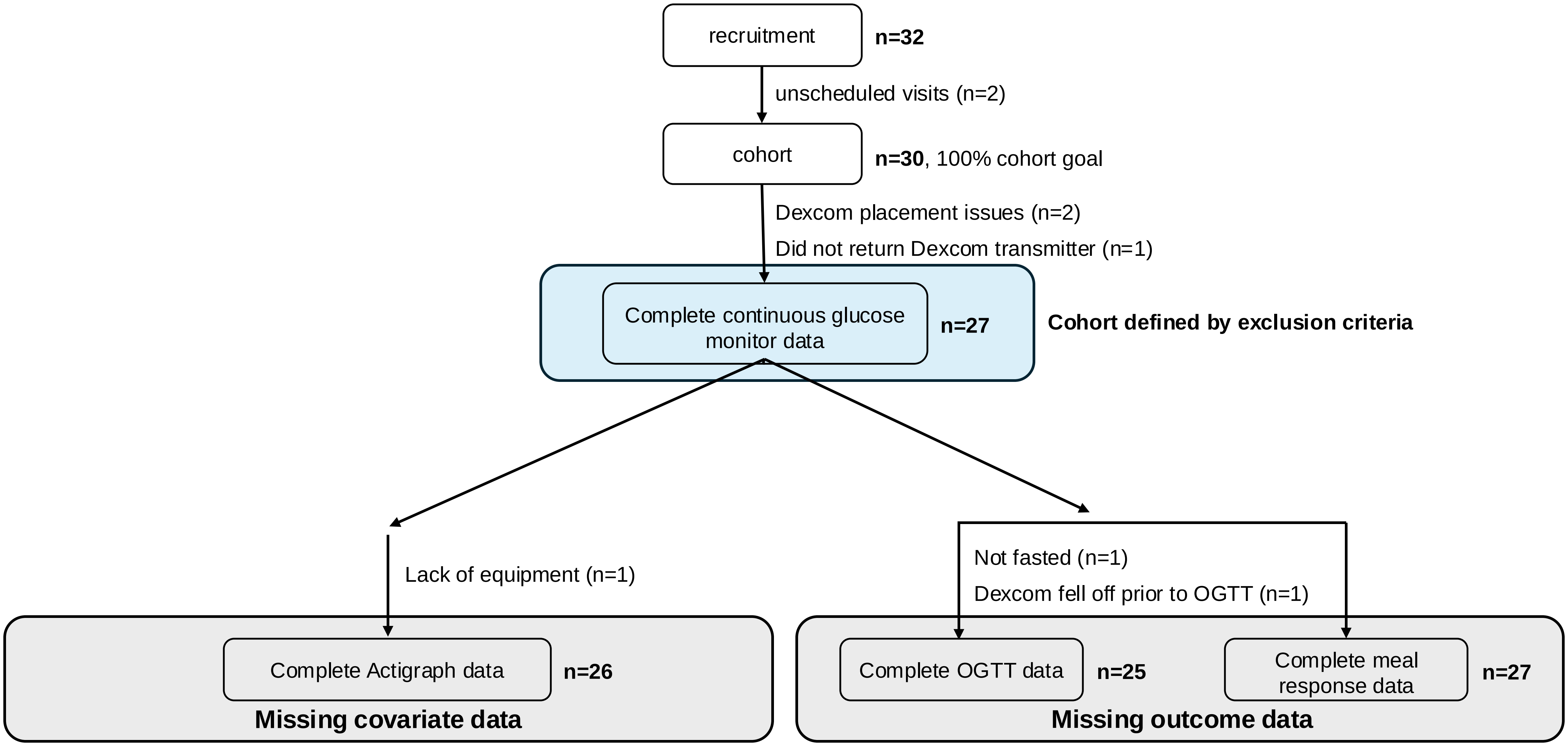

Supplementary Figure 2. Data availability within study cohort. Thirty participants completed Visit 1 and 2, with three participants excluded due to inability to collect Dexcom G6 continuous glucose monitors. The study cohort was defined with these twenty-seven participants. Twenty-six participants had complete Actigraph data. Twenty-five participants had completed the at-home oral glucose tolerance test while wearing the Dexcom G6 monitor. All twenty-seven participants had three 24-hour dietary recalls reported.

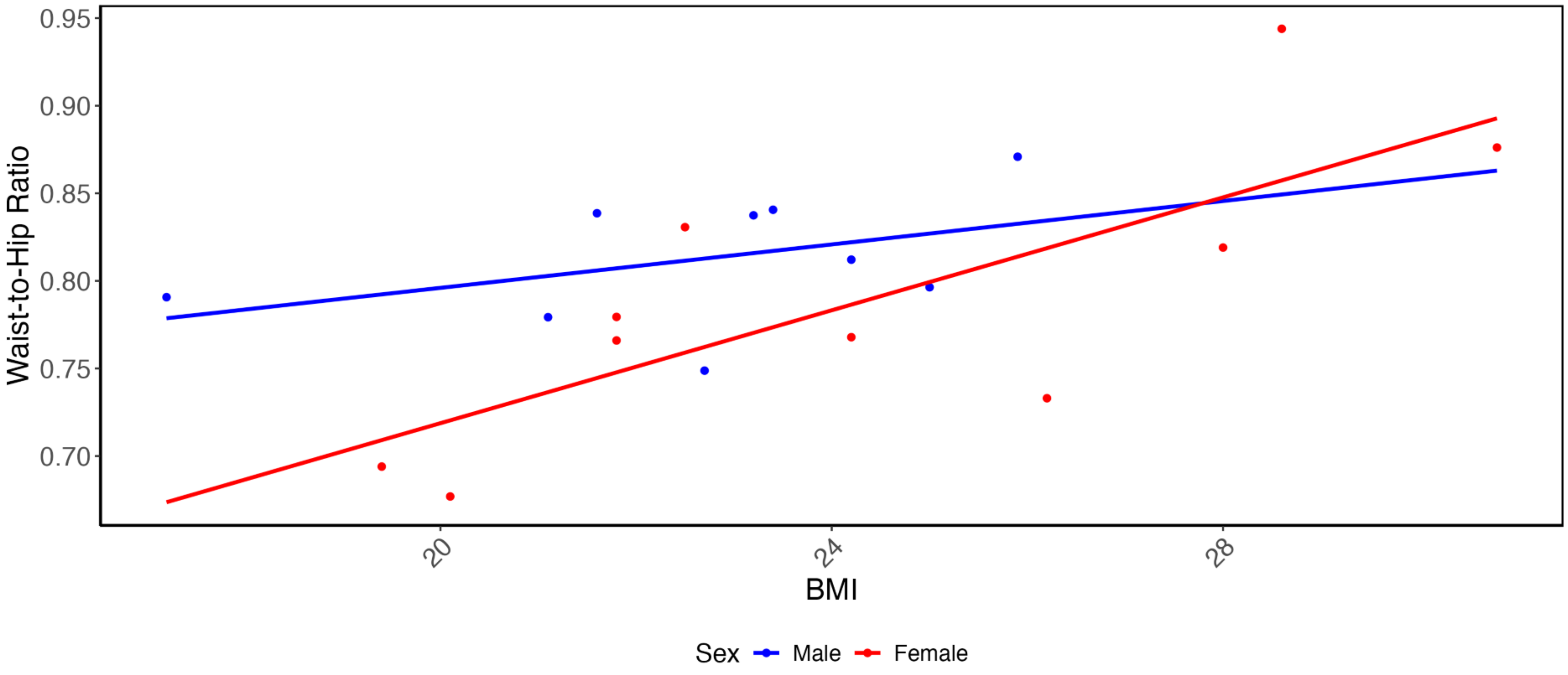

Supplementary Figure 3. Relationship between body mass index (BMI) and waist-to-hip ratio, stratified by sex. A positive correlation is observed between BMI (kg/m^2) and waist-to-hip ratio, exhibiting a stronger collection in females (Spearman correlation, r=0.76) than males (Spearman correlation, r=0.54).

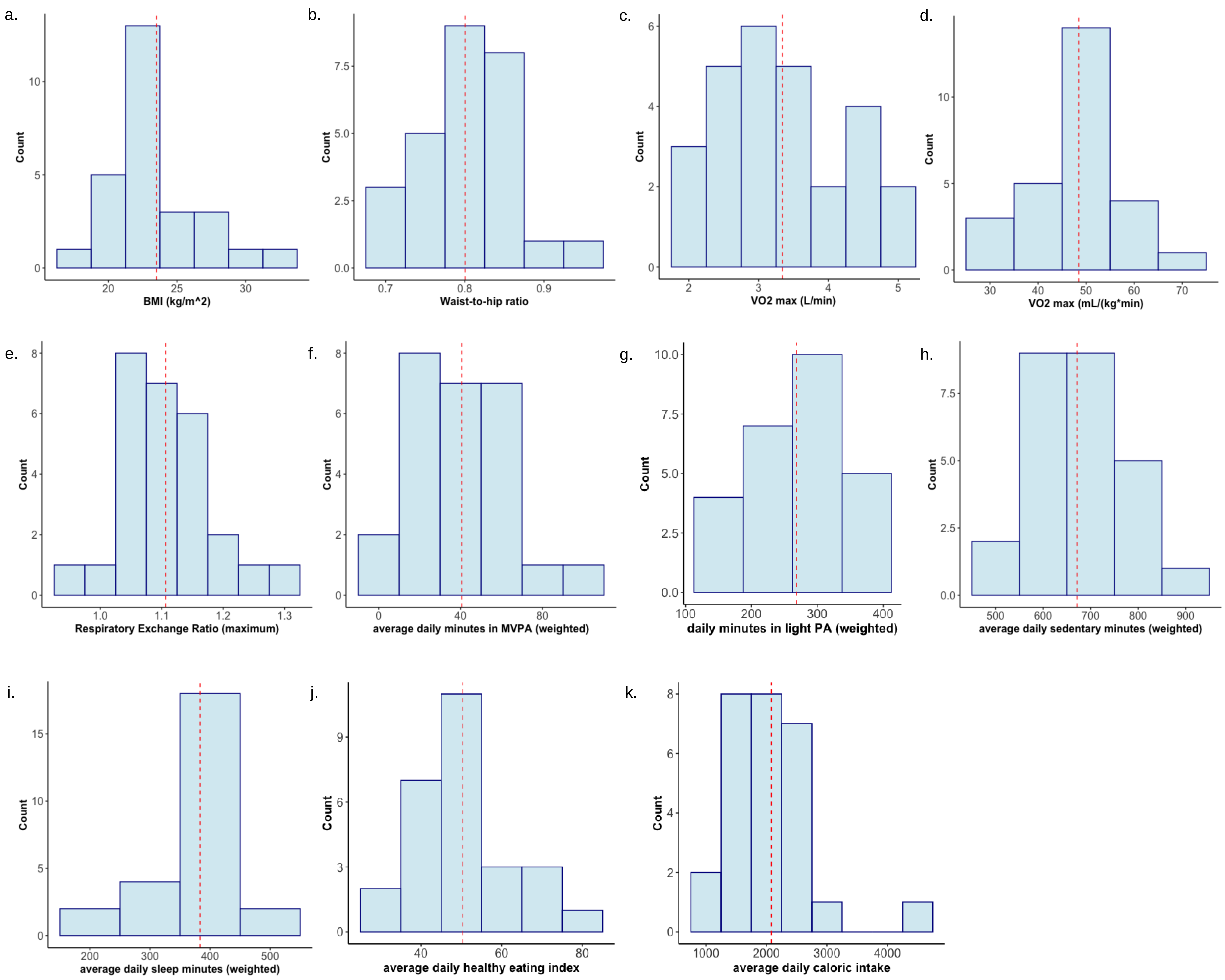

Supplementary Figure 4. Histograms of physical health and lifestyle characteristics. (a) BMI (kg/m^2); (b) waist-to-hip ratio; (c) VO2 max (L/min) (d) VO2 max, adjusted for weight (mL/(kg*min)); (e) respiratory exchange ratio (maximum reached); (f) average daily minutes in moderate-to-vigorous activity (weighted); (g) average daily minutes in light activity (weighted); (h) average daily minutes in sedentary (weighted); (i) average daily minutes sleeping (weighted); (j) average healthy eating index; and (k) average caloric intake (kcal). Red line represents mean.

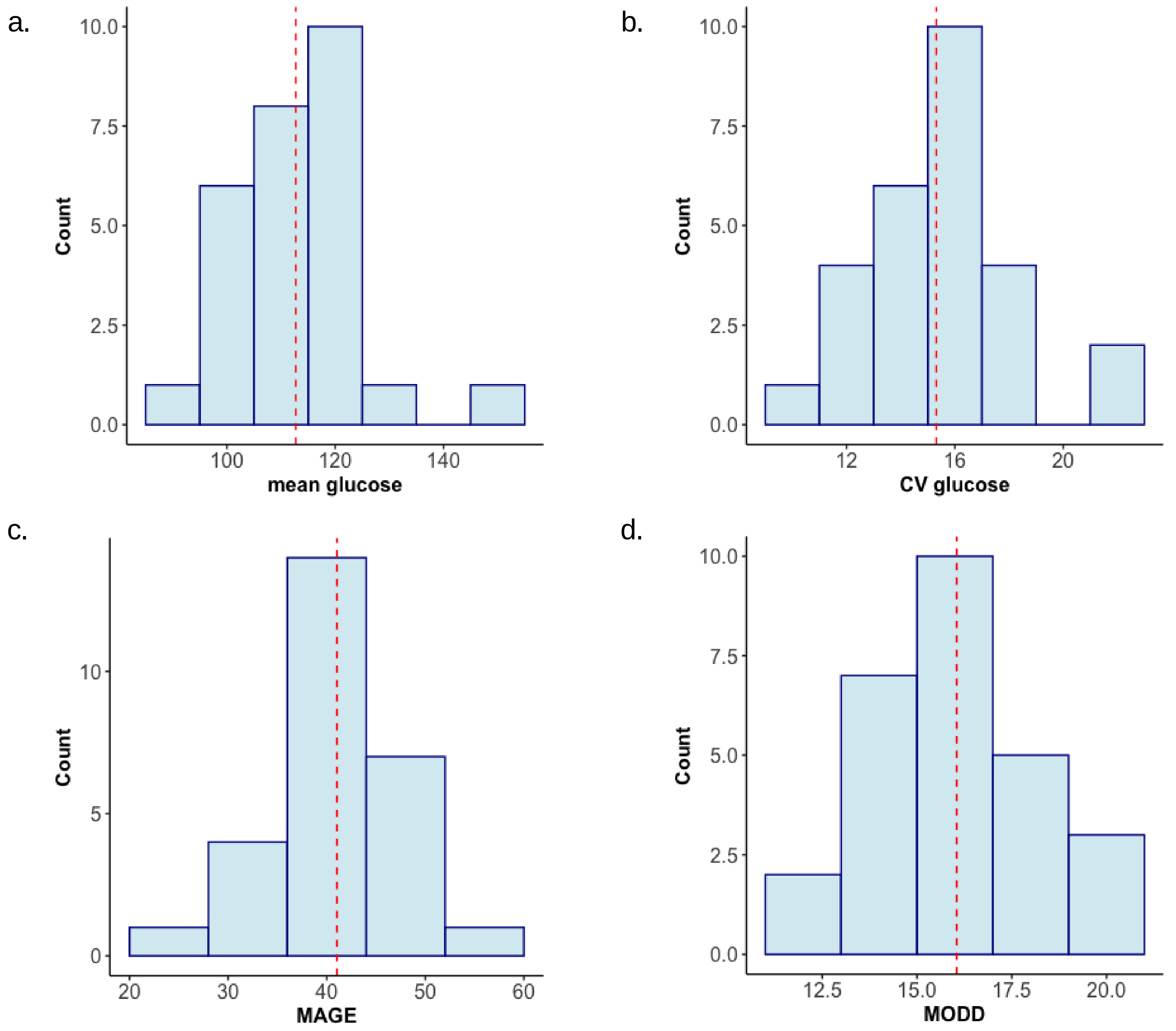

Supplementary Figure 5. Histograms of glucose metrics extracted from continuous glucose monitor. Glucose metrics extracted utilizing iGlu (n=27). (a) Mean glucose levels across all continuous glucose monitor measures; (B) coefficient of variance across all continuous glucose monitor measures; (c) mean amplitude of glycemic excursions; and (d) mean of daily differences. Red line represents mean.

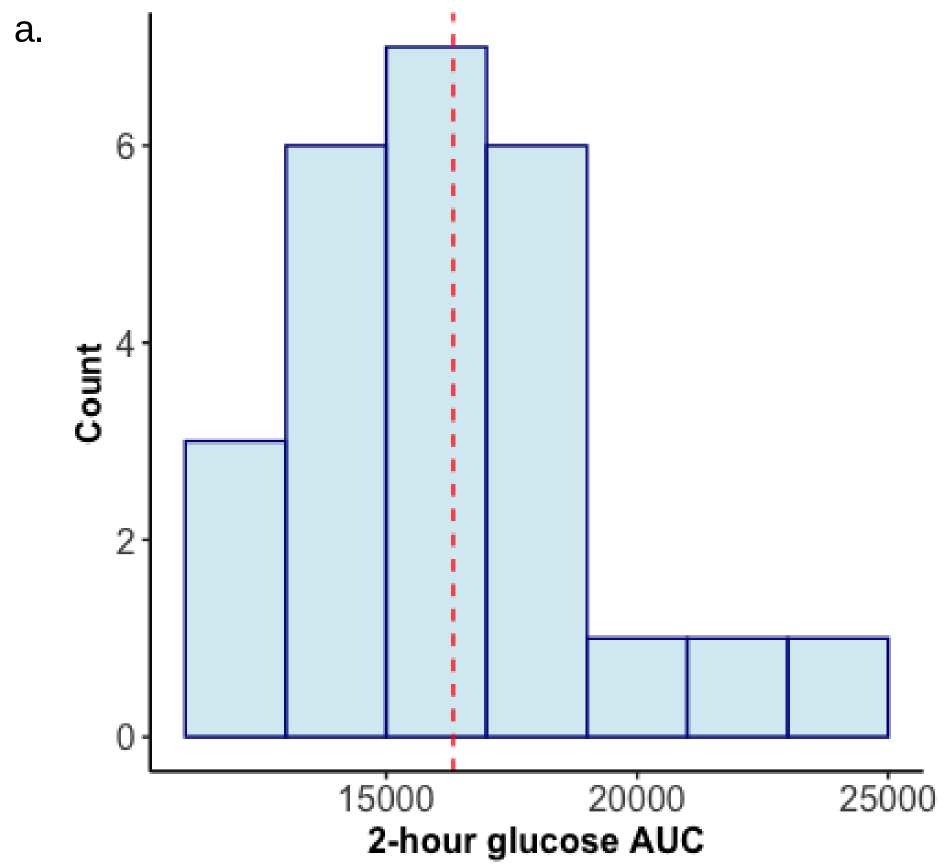

Supplementary Figure 6. Histograms of glucose response to oral glucose tolerance test. Two-hour glucose response to oral glucose tolerance test (n=25) reported as (a) glucose AUC and (b) max glucose. Red line represents mean.

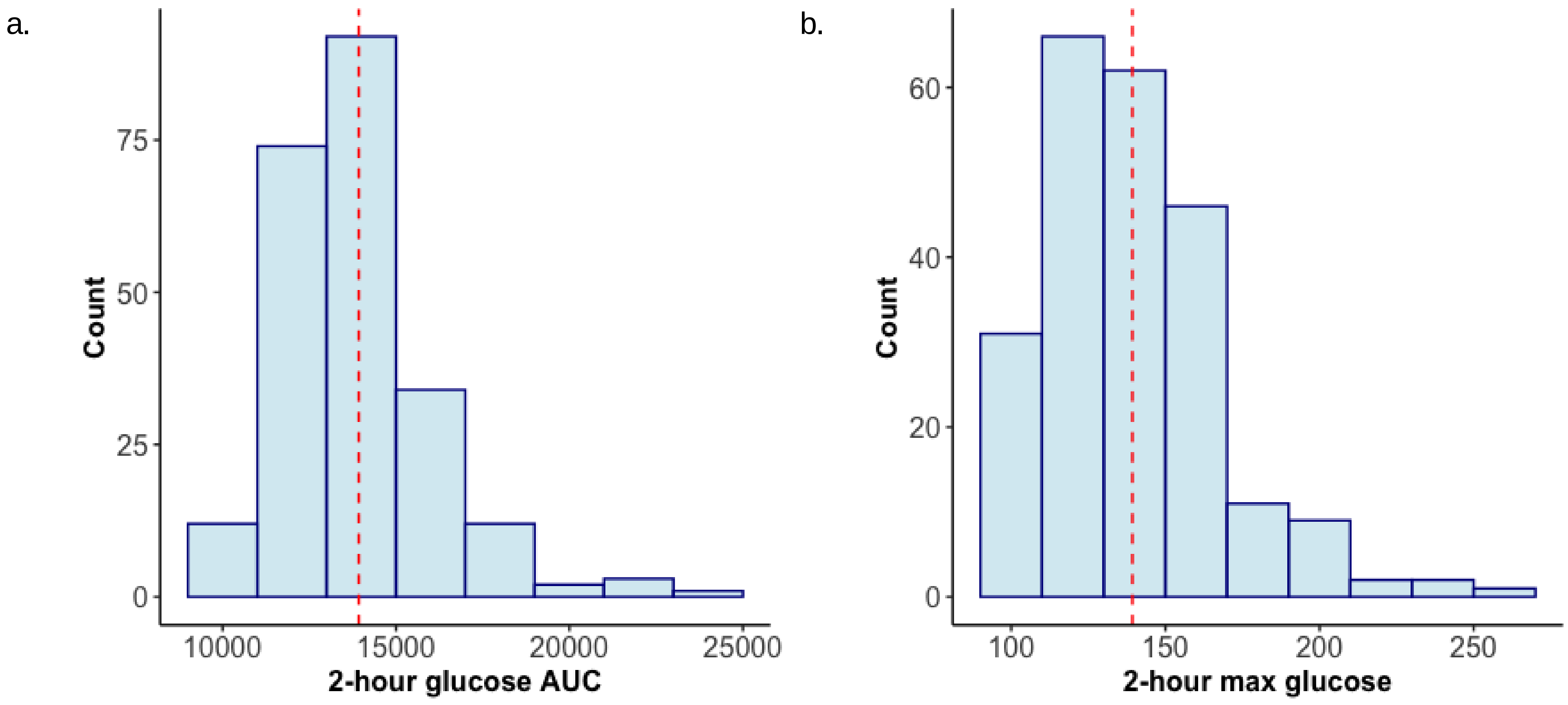

Supplementary Figure 7. Histograms of glucose response to individual meals. Two-hour glucose response to habitual dietary intake (230 meals) reported as (a) glucose AUC and (b) max glucose. Red line represents mean.

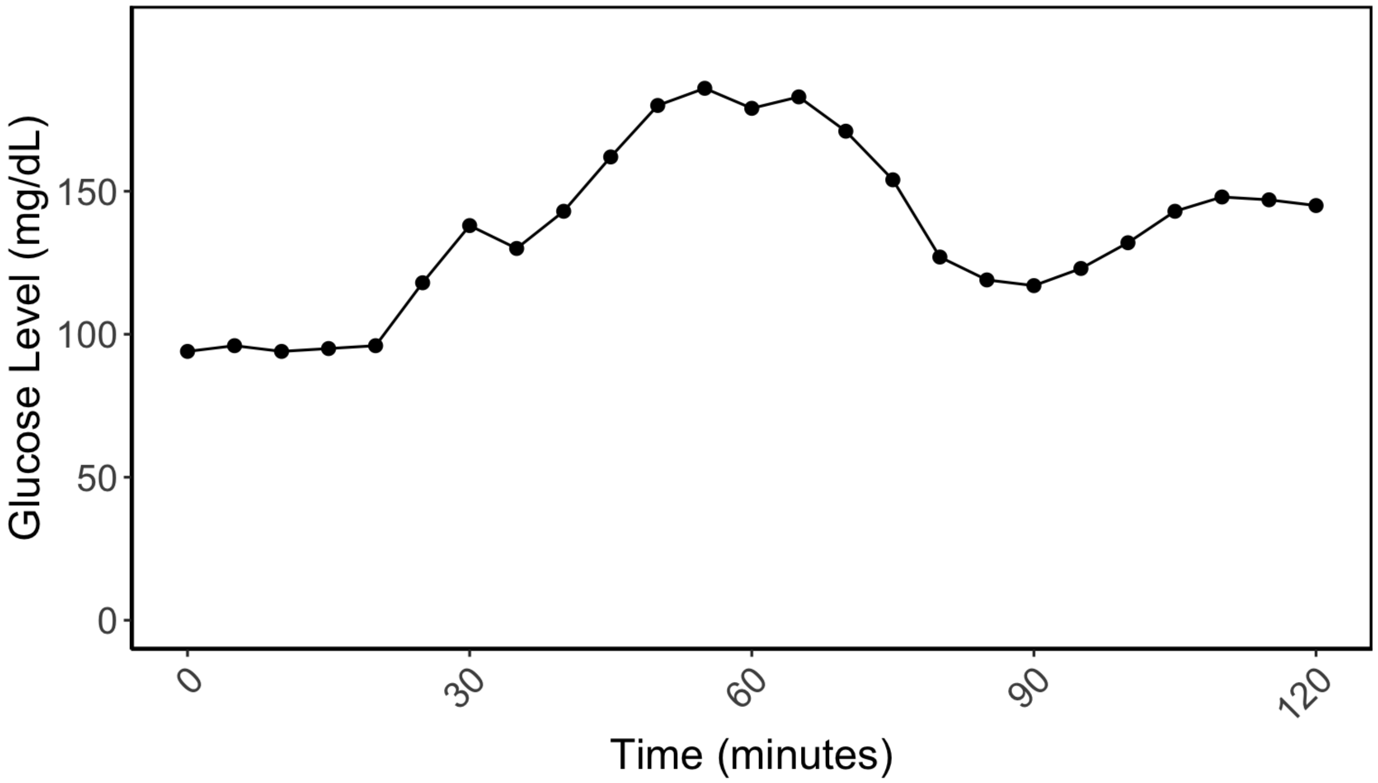

Supplementary Figure 8. Individual response to the at home glucose tolerance test. Participant 004 reported consuming the glucose tolerance test at 9:00am. Glucose levels did not rise from baseline until 26 minutes after the self-reported start time.
